# Ancient DNA SNP-panel data suggests stability in bluefin tuna genetic diversity despite centuries of fluctuating catches in the eastern Atlantic and Mediterranean

**DOI:** 10.1038/s41598-021-99708-9

**Published:** 2021-10-20

**Authors:** Adam J. Andrews, Gregory N. Puncher, Darío Bernal-Casasola, Antonio Di Natale, Francesco Massari, Vedat Onar, Nezir Yaşar Toker, Alex Hanke, Scott A. Pavey, Castrense Savojardo, Pier Luigi Martelli, Rita Casadio, Elisabetta Cilli, Arturo Morales-Muñiz, Barbara Mantovani, Fausto Tinti, Alessia Cariani

**Affiliations:** 1 Department of Biological, Geological and Environmental Sciences, University of Bologna, Ravenna, Italy; 2 Department of Cultural Heritage, University of Bologna, Ravenna, Italy; 3 Department of Biological Sciences, Canadian Rivers Institute, University of New Brunswick, Saint John, NB Canada; 4 Department of History, Geography and Philosophy, Faculty of Philosophy and Letters, University of Cádiz, Cádiz, Spain; 5 Aquastudio Research Institute, Messina, Italy; 6 Osteoarcheology Practice and Research Centre and Faculty of Veterinary Medicine, Istanbul University-Cerrahpaşa, Avcılar, Istanbul, Turkey; 7 St. Andrews Biological Station, Fisheries and Oceans Canada, St. Andrews, NB Canada; 8 Biocomputing Group, University of Bologna, Bologna, Italy; 9 Department of Biology, Autonomous University of Madrid, Madrid, Spain; 10 Department of Biological, Geological and Environmental Sciences, University of Bologna, Bologna, Italy

**Keywords:** Ecological genetics, Evolutionary ecology, Marine biology

## Abstract

Atlantic bluefin tuna (*Thunnus thynnus*; BFT) abundance was depleted in the late 20th and early 21st century due to overfishing. Historical catch records further indicate that the abundance of BFT in the Mediterranean has been fluctuating since at least the 16th century. Here we build upon previous work on ancient DNA of BFT in the Mediterranean by comparing contemporary (2009–2012) specimens with archival (1911–1926) and archaeological (2nd century BCE–15th century CE) specimens that represent population states prior to these two major periods of exploitation, respectively. We successfully genotyped and analysed 259 contemporary and 123 historical (91 archival and 32 archaeological) specimens at 92 SNP loci that were selected for their ability to differentiate contemporary populations or their association with core biological functions. We found no evidence of genetic bottlenecks, inbreeding or population restructuring between temporal sample groups that might explain what has driven catch fluctuations since the 16th century. We also detected a putative adaptive response, involving the cytoskeletal protein synemin which may be related to muscle stress. However, these results require further investigation with more extensive genome-wide data to rule out demographic changes due to overfishing, and other natural and anthropogenic factors, in addition to elucidating the adaptive drivers related to these.

## Introduction

Overfishing has reduced numerous fish populations to remnants of their historical levels^1,2^, yet we have a poor understanding of what impact this has had on their evolutionary potential and resilience^3^. This information is crucial to predict future demographic changes and thus promote sustainable fisheries management^4,5^. Studies of historical marine ecology offer an opportunity to learn and heed these past lessons^5–7^. In particular, genetic/genomic studies can infer past history from contemporary samples^8^, or directly test archaeological and archival samples^9^ for losses in genetic diversity, population restructuring, or adaptive responses to natural factors e.g., climate, or anthropogenic ones e.g., fisheries-induced evolution (FIE)^10^. A decade ago, Riccioni et al.^11^ were the first to investigate temporal demographic changes in the key species Atlantic bluefin tuna (*Thunnus thynnus*, hereafter BFT) using archival early-20th century samples and microsatellite markers. Here, we build on this work by genotyping archival and archaeological samples to extend investigations into the pre-industrial era, when fishing may have also had the potential to impact BFT.

BFT is a highly migratory pelagic top predator, characterized by its large size (up to 3.3 m in length and 725 kg in weight), slow maturation (between 4 and 8 years)^12,13^, and inshore migration behaviour, that has made it vulnerable to overfishing. Recent genomic studies^14,15^ support the delineation of two BFT populations. These are a western Atlantic component that spawns predominantly in the Gulf of Mexico^16^, and an eastern Atlantic and Mediterranean component that spawns predominantly in the Mediterranean Sea^17^. Individuals of both populations migrate into the Atlantic Ocean to feed, including as juveniles^18^, and exhibit high-levels of mixing^14,15^. The role of additional contemporary and historical spawning areas i.e. the Slope Sea (East of Cape Hatteras, USA)^16^, the Bay of Biscay^19^, and the Black Sea^20,21^, are yet to be clearly defined, especially regarding the Slope Sea where connectivity between populations was observed^15^.

During the last few years, the eastern Atlantic and Mediterranean population of BFT has recovered to 1970’s levels following heavy overfishing that depleted spawning stock biomass, restructured the population toward younger individuals, and contracted the species range, in the late 20th and early 21st century^22–25^. However, reconstructions of 16th-20th century BFT trap catch records suggest abundance across the Mediterranean has been fluctuating for centuries^23,26,27^. Pelagic species are particularly susceptible to fluctuations in abundance since dynamic food and environmental conditions drive large variability in recruitment, but fishing magnifies poor recruitment and therefore population declines when large catches occur^28^. Multiple factors need to be taken into account to be able to interpret trap catch fluctuations as abundance^29^, though, it appears that catch numbers in the 16th and 18th century may be comparable to those during the industrial fishing of the last 50 years^26,27^. Hence, fishing appears to have been intense in this period.

The current study investigates genetic variability in eastern Atlantic and Mediterranean BFT prior to both their 21st century population collapse, and record trap catches in the 16th and 18th century, using archived early-20th century specimens, and archaeological remains, respectively. Despite overfished species having an overall lower genetic diversity when contemporary data are compared^3^, Riccioni et al.^11^ were unable to detect losses in BFT genetic diversity when comparing contemporary and early-20th century samples. Likewise, no genetic erosion was observed following overfishing in the closely-related albacore (*Thunnus alalunga*)^30^, the Pacific herring (*Clupea pallasii*)^31^, or the European anchovy (*Engraulis encrasicolus*)^32^. Even marine species (e.g., sawfish, *Pristis* spp.) depleted to between 1 and 5% of their historical biomass appear to have retained genetic diversity^33^. However, several studies have noted genetic diversity declines or population losses following overfishing in Atlantic cod (*Gadus morhua*)^34–36^, Atlantic salmon (*Salmo salar*)^37^, and Chinook salmon (*Oncorhynchus tshawytscha*)^38^. In addition, adaptive responses to size- (in Walleye, *Sander vitreus*)^39^, and sex- (in Atlantic salmon)^40^ selective harvesting, and environmental drivers (in Atlantic cod)^41^ have also been reported in studies using archival or archaeological fish samples. It remains unclear to what extent the inability of some studies to detect these differences results from the selection of genetic markers with low resolution, or the resilience offered by complex life history traits during times of population decline. A recent whole genome sequencing (WGS) study^42^, that did not detect genetic erosion or adaptive responses in two Atlantic cod populations following 20th century overfishing, may indicate, however, that the latter is the case for some populations.

Here we test the hypotheses that the genetic diversity of BFT has declined and that their populations restructured following periods of intense fishing in the eastern Atlantic and Mediterranean. Further, we sought to identify adaptive responses that may be related to ecological or environmental conditions. To this end, our objectives were to genotype archaeological and archival specimens on a single nucleotide polymorphic (SNP) panel to (1) characterise their genetic diversity and population structure, (2) to compare those patterns to analogous ones from contemporary groups, and (3) to explore markers under putative selection and identify their associated function, if possible.

## Methods

### Samples

**Figure 1:**
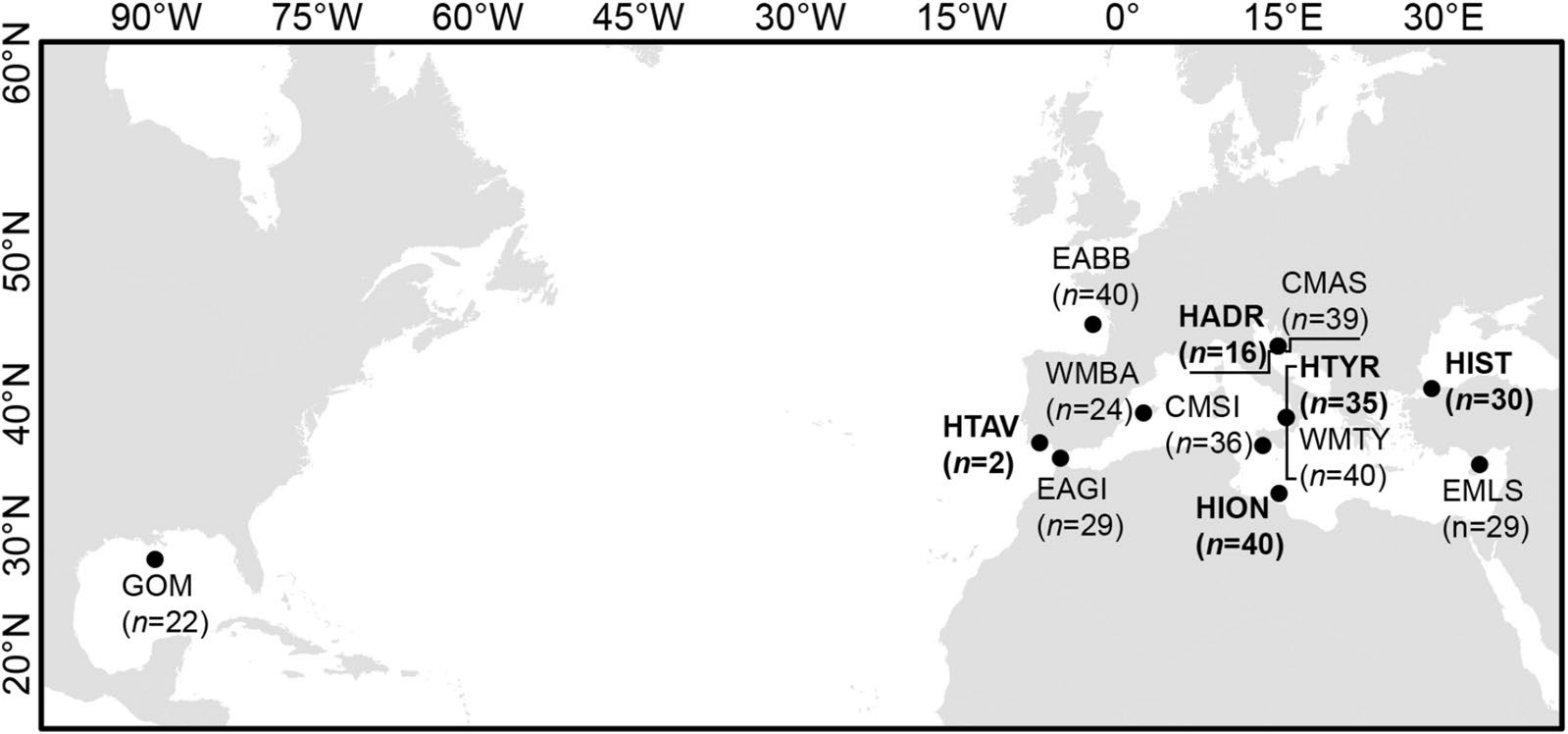
Map of the collection location for samples used in analyses. Historical (archival and archaeological) sample groups (in boldface, denoted with H) use approximate locations and the locations of archaeological sites where fish remains were recovered. Map created using ESRI ArcMap (v.10.6, https://arcgis.com). Only sample groups that were successfully genotyped and analysed are displayed. Numbers (*n*) represent those included in the final analysis for each sample group.

We collected samples of contemporary, archival and archaeological BFT specimens for analysis as follows: Contemporary reference specimens (GOM: Gulf of Mexico, CMAS: Central Mediterranean Adriatic Sea, CMSI: Central Mediterranean Sicily, EABB: East Atlantic Bay of Biscay, EAGI: Eastern Atlantic Gibraltar, EMLS: Eastern Mediterranean Levantine Sea, WMBA: Western Mediterranean Balearic Islands, WTYR: Western Mediterranean Tyrrhenian Sea, n = 277, Table S1) at each life stage were collected across the species range between 2009 and 2012 (Fig. 1, Table S1) where tissue samples from each specimen were preserved in 96% ethanol or RNAlater (Thermo Fisher Scientific, USA) and stored at − 20 °C until further processing. Archived vertebrae (HBOS: Historical Bosporus, HADR: Historical Adriatic Sea, HION: Historical Ionian Sea, HTYR: Historical Tyrrhenian Sea: n = 147, Table S1) from the Massimo Sella Archive (see^11^) were collected between 1911 and 1941 (Fig. 1). Archaeological vertebrae (n = 136, Table S1) were retrieved from several excavations (Fig. 1) including 4th–15th century CE Yenikapi (HIST: Historical Istanbul, Turkey)^43^, 2nd century BCE–5th century CE Baelo Claudia (HBC: Historical Baelo Claudia, Spain)^44^, 2nd century BCE Tavira (HTAV: Historical Tavira, Portugal), and 4th–2nd century BCE Palacio de Justicia, (HPJ: Historical Palacio de Justicia, Spain)^45^. See Supplementary Materials 1 for more details on historical samples and their dating.

### Contemporary DNA extractions

DNA was isolated from fin (adults) or muscle (juveniles, young-of-the-year) of contemporary samples (Table S1) as part of another study^14^ using the Wizard^®^SV96 Genomic DNA Purification Kit (Promega, USA), following the manufacturer's instructions. Quantification was performed using a NanoDrop 2000 (Thermo Fisher Scientific, USA). Negative controls indicated that no cross-contamination took place between samples.

### Ancient DNA extractions

Archival and archaeological samples underwent ancient DNA (aDNA) extraction in sterile, PCR-free conditions at the Ancient DNA Laboratory of the Department of Cultural Heritage (University of Bologna, Ravenna Campus, Italy), as part of another study which investigated their species identification via barcoding^46^. All bone specimens were sprayed with 1–2% sodium hypochlorite (bleach), left to soak for ten minutes, rinsed with distilled water and air-dried (as per^47^). Specimens were then mechanically cleaned using sandpaper, and the bleaching process was repeated. After, each specimen was exposed to UV light (254 nm) for 15 min before drilling to obtain ~ 200 mg bone powder. Bones that were too small for drilling were bisected, and their inner matrices were crushed.

Isolation of aDNA was performed using a modified version of Dabney et al.^48,49^. Briefly, ~ 200 mg of bone powder from each sample was divided in two and placed into separate tubes. After an overnight incubation in EDTA (0.5 M, pH 8.0) and proteinase K, lysates (1000 µl) of each sample were pooled and combined with 3000 µl binding buffer composed of guanidine thiocyanate (5 M), Tween 20 (0.05%), isopropyl alcohol (40% v/v), sodium acetate (90 mM, pH 5.2), and distilled water. This mixture was then centrifuged through a MinElute spin column (Qiagen, Germany), and washed twice with 720 µl PE buffer, before a final elution in 60 µl of distilled water.

The total DNA obtained from each extraction was quantified using a Qubit^®^ dsDNA HS (High Sensitivity) Assay Kit (Thermo Fisher Scientific, USA). Negative controls employed for each batch of samples extracted indicated an undetectable level of contamination (< 500 pg/ml).

### DNA genotyping

A total of 273 contemporary samples, and 280 historical (145 archival and 135 archaeological) samples contained sufficient quantities of DNA (100 ng total) for genotyping (Table S1). Samples were genotyped using a 96 SNP-panel we developed from SNP’s identified by two studies^14,30^ that were polymorphic between contemporary sample groups (see^14^) or matched with gene functions. To identify protein association we blasted the flanking regions of these loci against sequences for Atlantic cod^50^, sea bass (*Dicentrarchus labrax*)^51^, BFT^52,53^ and an umbrella set of teleost sequences, on NCBI GenBank (https://blast.ncbi.nlm.nih.gov/Blast.cgi, Table S2) using the blastn option. Queries were considered matches if alignment coverage was > 80% and identity scores were > 80% (Table S2).

SNP genotyping was conducted first using Fluidigm 96.96 Dynamic Array™ Integrated Fluidic Circuits (Probes: SNPtype-FAM:SNPtype-HEX, Passive reference: ROX) on the BioMarkHD034 platform (SGIKER, Spain). Historical samples were re-genotyped at a second facility using the Fluidigm EP1 platform (ABL, Bedford Institute of Oceanography, Canada) to assess genotype error rates. Genotyping employed two negative controls for each run, which confirmed no cross-contamination, and three positive controls (CMAS01, CMAS02, CMAS03), reporting identical genotypes. Similarly, 21 (7.5%) historical samples were extracted and genotyped twice and reported acceptable replicates at 97.8 ± 3.6% accuracy.

### Quality control filtering

Prior to analyses, two loci (SNP85, SNP86, Table S2) with low call rates (98–100% missing data) were discarded. Individuals (148 out of 553, 26.7%) and two further loci (SNP45, SNP79) that contained > 10% missing data were then removed. Inconsistencies between the two facilities at the remaining 92 loci were then assessed. The remaining 146 historical individuals were subject to further filtering whereby they were removed if their genotypes were inconsistent between the two facilities at > 5% of loci. This removed a further 21 (14.4%) historical individuals achieved an overall genotyping success of 98.8% at 92 loci. Sample groups that contained a single individual as a result of filtering (HBOS, Table S1) were also removed. Historical duplicate samples resulting from the potential sampling of two or more bone specimens of the same individual were identified and removed by applying the function clonecorrect in the Poppr package^54^ as implemented in R v.4.0.3^55^. A single clone was evident in the HIST archaeological sample group (Table 1).

**Table 1:** Genetic diversity, Hardy–Weinberg deviation, and F_IS_ in contemporary and historical (archival and archaeological) sample groups.

	Contemporary								Historical				
	GOM	CMAS	CMSI	EABB	EAGI	EMLS	WMBA	WMTY	HADR	HION	HTYR	HIST	HTAV
N	22	39	36	40	29	29	24	40	16	40	35	30	2
aR^a^	177	178	178	178	178	176	176	177	175	177	177	177	139
H_e_^a^	0.346	0.369	0.364	0.359	0.369	0.367	0.369	0.365	0.380	0.358	0.362	0.367	0.353
H_o_^a^	0.332	0.358	0.358	0.344	0.326	0.372	0.377	0.340	0.357	0.344	0.355	0.353	0.305
P_HW_	0.936	0.932	0.973	0.980	1.000	0.197	0.155	1.000	0.977	0.992	0.902	0.971	0.956
F_IS_^a^	0.032	0.024	0.033	0.034	0.137	− 0.018	− 0.022	0.066	0.052	0.041	0.023	0.038	–

n, number of samples analysed, aR, allelic richness; H_e_, mean expected heterozygosity; H_o_, mean observed heterozygosity; P_HW_, P value of the Hardy–Weinberg equilibrium deviation test; F_IS_, inbreeding coefficient.

^a^All unpaired t-test values between contemporary and historical samples (excluding GOM, HTAV) were non-significant.

### Loci evaluation

Deviation from Hardy–Weinberg equilibrium (HWE) was assessed at each locus using the R package Pegas^56^. Linkage disequilibrium (LD) between loci was tested using the R package Genepop^57^. Outlier loci were identified using Bayescan^58^ and OutFLANK v0.2^59^ to obtain a neutral dataset and identify potential adaptive responses. Analysis was run excluding the western Atlantic sample group (GOM) between the following: all sample groups, pooled contemporary and historical sample groups, contemporary sample groups, and historical sample groups. Loci detected as outliers were removed from the dataset prior to demographic analyses and investigated as follows: gene association was inferred from the above blastn searches, and non-synonymous mutations were explored with the Expasy Translation tool as implemented online (https://web.expasy.org/translate). Default settings were used in the analyses. Significance was judged using the False Discovery Rate (FDR)^60^ approach at the 5% level, as calculated using 999 permutations.

### Genetic diversity

Allelic richness (aR), heterozygosity expected/observed (H_e_, H_o_), and the inbreeding coefficient F_IS_, were calculated for each sample group with the R package Hierfstat^61^. Significance of heterozygote excess was calculated with Genepop in R using the global excess method and default settings. Differences in aR, H_e_, H_o_ and F_IS_ between pooled contemporary and historical sample groups were assessed using unpaired t-tests in R. Significance was judged at the 5% level. Effective population size (N_e_) estimates were calculated only for samples consistently scored across all 89 neutral loci, as summarised in Table 2. Estimations were calculated using the linkage disequilibrium approach^62^ as implemented in N_e_Estimator v2.1^63^ and an allele frequency threshold of 0.01. A random down-sampling to generate and analyse equal-size groups is summarised in Table S3. Because N_e_ estimates are often unreliable at low sample sizes^64^, we calculated per locus round-robin estimates of minor allele frequencies in R (as per^65^) and plotted trajectories between temporal sample groups. We performed a hierarchical analysis of molecular variance (AMOVA) in Poppr, with 10,000 permutations to assess significance. AMOVAs were performed excluding the GOM sample group on the following levels: between periods; between sample groups; between samples (i.e., individuals); and within samples.

**Table 2:** Effective population size (N_e_) and 95% confidence Intervals of contemporary and historical sample groups for samples (n) consistently scored across all 89 neutral loci analysed herein, under two approaches, where separate estimates were made for each sample group and for contemporary and historical pools.

	Contemporary								Historical			
	GOM	CMAS	CMSI	EABB	EAGI	EMLS	WMBA	WMTY	HADR	HION	HTYR	HIST
**Separate**												
n	14	32	27	30	–	14	16	36	–	24	24	18
N_e_	140	164	391	1454	–	2515	140	150	–	825	60	14
CI	56 − ∞	78–799	111 − ∞	158 − ∞	–	68 − ∞	46 − ∞	53 − ∞	–	118 − ∞	40–114	11–18
**Pooled**												
n	0	32	27	30	4	14	16	36	4	24	24	18
N_e_	939								298			
CI	497–5465								178–787			

### Population structure

A discriminant analysis of principal components (DAPC) was performed with the R package Adegenet^66^ to explore how the historical groups relate to the contemporary reference groups. DAPC is a geometric clustering method free of HWE and LD assumptions, that attempts to maximise the inter-variation between clusters while minimising the intra-variation observed within clusters. DAPC clusters were set a priori to the number of sample groups. We retained 4 discriminant functions and the number of principal components (PC’s) according to the function optim.a.score, based on an initial selection of all PC’s before refinement. Population structuring was also evaluated using STRUCTURE v.2.3.4^67^, which implements a Bayesian clustering method to identify the most likely number of populations (K). We followed the Evano et al.^68^ method, and thus, we carried out 10 runs per each value of K ranging from 1 to 10. Runs used the locprior and admixture models and assumed correlated allele frequencies. Each run used 500,000 burn-in and Markov Chain Monte Carlo replicates. We estimated the ad hoc statistic ΔK in order to infer the most likely number of populations using STRUCTURE HARVESTER^69^. CLUMPAK^70^ was used to merge the 10 runs from the most probable K, and reported similarity scores > 95. We used a hierarchical approach to improve resolution due to the identification of 5 outliers (EAGI 6 & 17, WMTY 52, 57 & 66) in two modern sample groups that constituted two distinct populations at K = 3 in the first run. Hence, these individuals were removed from the dataset and STRUCTURE was run a second time. Pairwise distances between sample groups were calculated with Nei's estimator of FST^71^ in the hierfstat R package, using 999 permutations to calculate the respective p-values, which were judged for significance under the FDR approach at the 5% level.

## Results

### Loci evaluation

Overall, 259 contemporary, and 123 historical (91 archival and 32 archaeological) samples were analysed at 92 loci (Table S1). No loci deviated from HWE or were in LD in more than a single population. BayeScan and OutFLANK both detected three loci (SNP41, SNP43 & SNP93, Table S1, Figure S1) as outliers. Loci SNP41 and SNP43 were outliers between contemporary sample groups and locus SNP93 was an outlier between historical sample groups. Locus SNP41 was identified as a putative adaptive response after being detected as an outlier between pooled contemporary and historical groups. Locus SNP41 was found to be in potential association with the gene SYNM that encodes Synemin, which is an intermediate filament protein. This putative adaptive locus was found to be under selection in all contemporary sample groups except CMSI, comprising a nucleotide mutation (T to A) that was non-synonymous, resulting in the production of glutamine instead of histidine. In contrast, SNP41 was not under selection in a single historical sample group.

### Genetic diversity

We found no significant differences in gene diversity aR (*p* = 0.181, t(11) = 1.426), H_e_ (*p* = 0.923, t(11) = 0.099) and H_o_ (*p* = 0.575, t(11) = 0.578) between pooled contemporary and historical groups (Table 1). Heterozygote deficiency was not significant in any sample group (Table 1). Inbreeding (F_IS_) was rare within all sample groups (Table 1) and was not significantly different between pooled contemporary and historical samples (*p* = 0.939, t(9) = 0.0791). The dataset lacked power to define reliable estimates of N_e_ using both methods for each sample group i.e., our CIs contained infinity until they were pooled (Table 2). Randomly excluding samples to create equal size sample groups had minimal influence on estimations (Table S3). N_e_ estimates were higher for both contemporary sample groups, analysed separately, and the contemporary eastern Atlantic and Mediterranean when pooled (Table 2). Allele trajectories (Figure S1) showed stochastic fluctuations in minor allele frequencies between all sample groups, and no consistent drop-out or over-estimation in all contemporary or historical sample groups, respectively. Within the eastern Atlantic and Mediterranean samples, AMOVAs indicated significant differences in variance within and between samples, and between sample groups, but not between periods (Table 3, Figure S2).

**Table 3:** Variance of the eastern Atlantic and Mediterranean BFT samples as computed by AMOVAs using a hierarchical approach as indicated by the four levels.

	Σ	% variance	*p* value
Within samples	31.203	96.010	< 0.001
Between samples	1.203	3.703	0.001
Between sample groups	0.087	0.268	0.003
Between contemporary and historical groups	0.005	0.017	0.306

### Population structure

**Figure 2:**
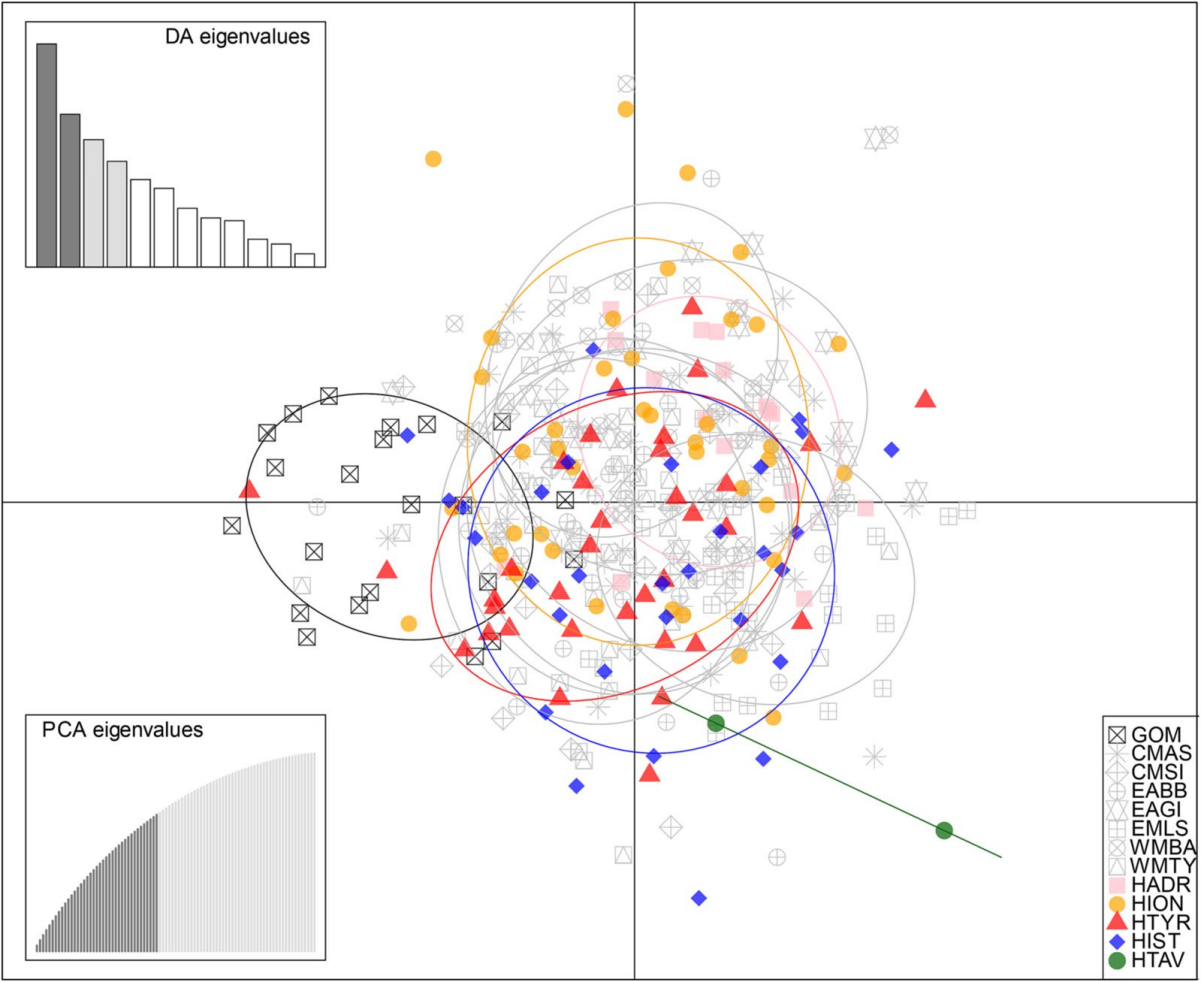
Discriminant analysis of principal components scatterplot showing how historical (archival and archaeological, denoted with H) sample groups relate to contemporary reference populations of the Gulf of Mexico (GOM) and the eastern Atlantic and Mediterranean. DAPC cluster ellipses were set to contain 95% of genotypes. Discriminant analysis (DA) eigenvalues and principal component analysis (PCA) eigenvalues were selected as displayed to avoid overfitting, utilising the optim.a.score approach within the R package adegenet.

**Figure 3:**
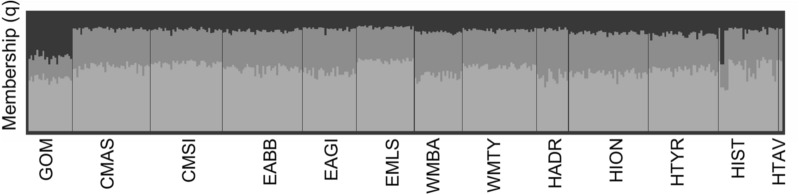
STRUCTURE barplot showing membership probabilities (q) for each sample group analysed herein with K = 3 (each represented by a different shade). K = 3 was the most likely number of populations identified by the ΔK method. Historical (archival and archaeological) sample groups are denoted with H).

DAPC clustered eastern Atlantic and Mediterranean sample groups together while the western Atlantic (GOM) sample group was substantially separated (Fig. 2). Considerable overlap was observed between contemporary and historical clusters of the eastern Atlantic and Mediterranean. ΔK suggested that the most likely number of populations identified with STRUCTURE was K = 3. All individuals shared mixed membership (q). Separate structuring of the GOM sample group was evident and the historical sample group HIST contained three individuals with this signature (Fig. 3). Overall, no evidence of population structure was evident between contemporary or historical sample groups of the eastern Atlantic and Mediterranean (Figs. 2, 3). Pairwise F_ST_ values were significant between the GOM sample group and all others (Table 4). In addition, the sample groups EMLS and WMBA, and EAGI and HIST were significantly different. No other significant differences were observed between contemporary and historical sample groups.

**Table 4:** Pairwise F_ST_ (below the diagonal) and non-corrected P values (above the diagonal) between contemporary and historical sample groups.

	Contemporary								Historical				
	GOM	CMAS	CMSI	EABB	EAGI	EMLS	WMBA	WMTY	HADR	HION	HTYR	HIST	HTAV
GOM		**0.001**	**0.001**	**0.001**	**0.001**	**0.001**	**0.001**	**0.001**	**0.001**	**0.001**	**0.001**	**0.001**	**0.001**
CMAS	0.0154		0.643	0.387	0.065	0.153	0.023	0.127	0.446	0.810	0.351	0.450	0.425
CMSI	0.0128	− 0.0009		0.566	0.053	0.448	0.045	0.364	0.0295	0.602	0.029	0.538	0.221
EABB	0.0118	0.0006	− 0.0004		0.108	0.111	0.174	0.416	0.749	0.635	0.593	0.625	0.702
EAGI	0.0240	0.0041	0.0046	0.0033		0.002	0.124	0.045	0.155	0.249	0.004	**0.001**	0.615
EMLS	0.0255	0.0027	0.0003	0.0034	0.0096		**0.001**	0.002	0.194	0.015	0.009	0.398	0.568
WMBA	0.0171	0.0064	0.0051	0.0029	0.0037	0.0109		0.116	0.689	0.273	0.007	0.085	0.103
WMTY	0.0167	0.0026	0.0007	0.0003	0.0047	0.0087	0.0035		0.099	0.022	0.197	0.597	0.413
HADR	0.0228	0.0004	0.0017	− 0.0025	0.0040	0.0037	− 0.0024	0.0048		0.853	0.248	0.876	0.211
HION	0.0129	− 0.0018	− 0.0006	− 0.0008	0.0017	0.0059	0.0016	0.0049	− 0.0040		0.217	0.562	0.238
HTYR	0.0117	0.0016	0.0053	− 0.0004	0.0083	0.0070	0.0087	0.0019	0.0026	0.0017		0.155	0.351
HIST	0.0155	0.0003	− 0.0003	− 0.0007	0.0097	0.0008	0.0042	− 0.0007	− 0.0047	− 0.0004	0.0027		0.594
HTAV	0.0350	0.0037	0.0183	− 0.0127	− 0.0079	− 0.0046	0.0322	0.0035	0.0201	0.0168	0.0085	− 0.0066	

*p* values that were significant after FDR correction are presented in boldface.

## Discussion

We found no evidence of genetic diversity loss or population restructuring in contemporary BFT sample groups of the eastern Atlantic and Mediterranean compared with those from the early 20th century CE prior to spawning biomass depletion and species range contraction^22,25^, and the 4th–15th century CE prior to a significant period of intense trap fishing marked by fluctuating catches^26,27^. If overfishing had resulted in a genetic bottleneck, we would expect to see significant decreases in minor allele frequencies, allelic richness, and observed heterozygosity^72^ for contemporary samples compared with historical samples. Therefore, we would also expect to observe an increase in inbreeding and a decrease in effective population size^8^, which we did not. The impact of overfishing on genetic diversity and allele frequencies has been observed in a variety of studies that directly test archaeological and archival samples^34–38^. At its most extreme, overfishing has been observed to restructure marine fish populations^36^, yet we found no evidence of genetic restructuring in BFT. Likewise, a recent study found that Atlantic cod had not been impacted by 20th century overfishing at the genomic level^42^. Our findings are similar to those of Riccioni et al.^11^ using microsatellite markers, though we did not observe significant sub-structuring within Mediterranean BFT as they did, and this is yet to be resolved to clarify alternative population structure hypotheses^18,23,73^. No recent genetic study, however, has detected population structure within the eastern Atlantic and Mediterranean BFT^14,15,74^. Perspectives from threatened populations of other taxa, inform us that a wide range of genomic responses are expected, along a continuous scale from resistance to collapse^34,75–79^, and recovery^33,80–82^. Despite differences between taxa, these data would suggest that there is likely no “one-size fits all” response to the depletion of marine fish populations, according to species life history traits and the extent and rate of overfishing.

The most common explanation for the maintenance of genetic diversity in threatened populations is that gene flow acts as a buffer^72^. This is plausible for BFT, though its western Atlantic population is smaller than the eastern Atlantic and Mediterranean population (ca. 1/10 the size) and was heavily fished itself since at least the early 19th century^83,84^. Connectivity with alternative spawning sites (e.g., the Slope Sea^16^, the Bay of Biscay^19^, Azores, Canary Islands, Ibero-Moroccan, Gulf of Guinea^13,17^) remains poorly understood, and the unresolved frequency and duration of spawning at these locations means we cannot assess its effect on gene flow. Likewise, introgression occurs at a low rate between *Thunnus* species^85^ but could also act as a buffer. On the other hand, eastern BFT may be resilient towards genetic erosion due to their relatively large population size (enhanced by connectivity between spawning sites within the Mediterranean), and a long life cycle which promotes heavily overlapping generations^13,17^. In any case, our findings leave us with two possible explanations; either (1) overfishing was not severe enough to cause a genetic bottleneck in BFT, or (2) our observation of significant demographic changes was hindered by the methods we employed.

To address this first point, it is evident that BFT were overfished, at least in the 20th and early 21st century, if not as we suspect between the 16th–19th century. Studies by the management body ICCAT (the International Commission for the Conservation of Atlantic Tunas)^25^, and independent estimates e.g.,^22^ suggested that BFT abundance and range declined by 70% and 46–53%, respectively, between 1960 and 2010. However, there is debate on the extent of the population decline, where on one hand, impending population collapse was predicted in 2009^86^, yet on the other, poorly understood population dynamics and incorrect assignments of catches has caused uncertainty in population estimates^73,87^. Hence, it is difficult to deduce whether we should expect to find evidence of a genetic bottleneck because the recent recovery of the population within just two generations from its lowest point in 2007^88^ could suggest that either the population decline was not that severe, or that overfishing did trigger a severe population decline but BFT is remarkably resilient due to its complex life history traits.

Nonetheless, fishing effort is not the only factor that influences catches and abundance (as shown for the historical trap fishery data^29^), which one might expect to be reflected in genetic diversity and structure. Climate is likely the largest regulator of recruitment and thus fish abundance^89,90^ and as a pelagic species, BFT are certainly no exception^91,92^. Therefore, one might expect to find evidence of fluctuating abundance—and potentially genetic diversity—that is merely exacerbated by fishing^28^. BFT’s Atlantic distribution varies with Atlantic multidecadal oscillation phases^93^, and thus gene flow and inbreeding is expected to vary accordingly because connectivity of populations is enhanced in warm years as ranges overlap, as attested by isotope data^94^. This is notwithstanding time-related effects driven by evolutionary processes i.e., mutation and genetic drift that we might expect to alter allele frequencies over time. Therefore, our observation of homogeneity between contemporary and historical BFT samples is somewhat striking. One might pose the question: at what rate should we expect to observe demographic changes at the genomic level? We analysed moderate sample sizes from 1911 to 1926 (~ 20 generations ago) and the 4th–15th century (~ 100 + generations ago), yet we did not detect time-related effects. Thus, to address this, even at conservative mutation rates lower than those shown for marine fish^95^, we would expect to observe changes in allele frequencies as a result of genetic drift alone.

Alternatively, our observations may be explained by our methodological approach. By pre-selecting loci that were polymorphic in contemporary sample groups, our data are subject to an unknown degree of ascertainment bias. Theoretically, ascertainment bias could influence any analysis or inference based on SNP allele frequencies when SNPs are discovered in a limited sample but applied in another context (e.g., our historical sample groups)^96^. The expectation that this should inflate diversity in the ascertainment sample is a widely accepted hindrance of SNP-panel studies^97–99^. Studies usually correct for this by LD pruning^100^ or modifying raw genotypes following maximum-likelihood simulations^98^, however this was not possible herein due to the few loci that were available. Indeed, the effect of ascertainment bias is likely to be exacerbated herein because we analysed few loci. This reduces the likelihood of detecting rare alleles and thereby erodes power^97^ which is particularly crucial when differentiating marine samples due to high gene flow and low diversity in marine populations^101^. Theoretically, this might have inflated our estimates of genetic diversity among contemporary samples, and hence genetic diversity was comparatively low in historical samples. This theory is further supported by our AMOVA results and might explain why variance was lower than expected between temporal samples, and why structure was only observed between contemporary sample groups for which SNP discovery was made.

Moreover, our N_e_ estimate CIs often contained infinity, suggesting that we have little power to make any inferences on N_e_. In many cases N_e_ was strikingly lower in (supposedly unimpacted) historical samples than the empirical rule-of-thumb threshold of N_e_ (500) proposed to maintain long-term genetic diversity in marine populations^72^. In any case, N_e_ is often unreliable when using sample sizes such as ours^64^ and we caution the interpretation of our results for this reason. Additionally, our sampling strategy may have been limiting. For example, if genetic diversity had decreased following population declines (e.g., between 16th–18th century, and/or during the 20th and early 21st century) but was restored prior to our analogous archival samples of the early 20th century, or 2009–2012 contemporary samples, respectively. Species differ in their rates of genetic recovery according to their life history traits^72^, and as this rate is unknown in BFT, we cannot rule out this possibility.

Clearly aDNA approaches offer utility to fisheries management because long-term trends are understudied and we lack fisheries-independent indices^7,90^. However, genome-wide approaches are more likely to provide a better resolution to assess demographic impacts and adaptive responses. Assuming the availability of a reference genome, WGS approaches are increasingly cost-effective^9^, particularly for shallow sequencing^102^. This approach may also facilitate the recovery of data from arid Mediterranean specimens which were challenging to genotype herein due to their poor preservation^46^. Importantly, WGS would reduce ascertainment bias compared with SNP-genotyping. This is crucial where allele frequency distributions are used to infer demographic history, but also to scan for past targets of selection^98^. We were limited herein to detecting a single adaptive response in BFT: a potential change to the function of the protein synemin, which is a cytoskeletal protein that we speculate might be related to growth changes induced by size selective harvesting (FIE), although this remains to be tested. WGS studies able to detect additional loci under putative selection are ultimately required for the association of this response (and others) with natural or anthropogenic factors, in addition to discounting hitchhiking effects^103^.

## Conclusion

We identify that aDNA preserved within archival and archaeological fish remains has the potential to inform fisheries management by providing novel fisheries-independent baselines with which to observe unstudied long-term demographic and adaptive changes. We found no evidence that genetic diversity decreased or that populations restructured following several centuries of intense fishing, in line with a previous study^11^. This may hint at BFT’s resilience which has been recently shown by rebounds in abundance^25^ and a return to previous habitats^88^. However, we acknowledge limitations in our dataset i.e., few markers and the potential for ascertainment bias, and suggest that future studies might benefit from obtaining WGS data to observe rare alleles and reduce bias. Genome-wide data will be especially necessary to investigate adaptive responses, such as the putative selection on the cytoskeletal protein synemin found herein, and associate these with natural or anthropogenic factors to elucidate the drivers of change.

## Supplementary Information

Supplementary Information 1.

Dataset S2.

Dataset S3.

## Data Availability

Flanking region sequences for each locus, and genotypes for all individuals, are attached as supplementary files.
