# Supplementary material for: Ancient DNA SNP-panel data suggests stability in bluefin tuna genetic diversity despite centuries of fluctuating catches in the eastern Atlantic and Mediterranean
Source: Sci Rep. 2021 Oct 20;11:20744. doi: 10.1038/s41598-021-99708-9 (PMC8528830; doi:10.1038/s41598-021-99708-9)
Supplement: Supplementary file 1 — Supplementary Information 1. [file 41598_2021_99708_MOESM1_ESM.docx]

## **SUPPLEMENTARY INFORMATION**

## **Ancient DNA SNP-panel data suggests stability in bluefin tuna genetic diversity despite centuries of fluctuating catches in the eastern Atlantic and Mediterranean**

Adam J. Andrews, Greg N. Puncher, Darío Bernal-Casasola, Antonio Di Natale, Francesco Massari, Vedat Onar, Nezir Yaşar Toker, Alex Hanke, Scott A. Pavey, Castrense Savojardo, Pier Luigi Martelli, Rita Casadio, Elisabetta Cilli, Arturo Morales-Muñiz, Barbara Mantovani, Fausto Tinti, Alessia Cariani

Supplementary Materials 1: Details of historical specimens analysed

1911-1941 CE Massimo Sella Archive

We analysed specimens collected from four separate locations in the early 20^th^ century by the ecologist Massimo Sella^[11](https://paperpile.com/c/8ySmCE/LIMRh)^. All specimens consist of vertebrae that were air-dried by the collator after capture and processing at tuna traps (*Tonnare*). A total of 50 samples were obtained from BFT vertebrae that were captured in 1911 close to the shore at Messina, Pizzo, Italy. These samples were considered to represent the Tyrrhenian Sea as a whole (HTYR). A total of 46 samples were obtained from BFT vertebrae captured in Zilten, Libya in 1926, considered to represent the Ionian Sea as a whole and named HION. A total of 49 samples were obtained from BFT vertebrae captured in the north of the Adriatic Sea in 1927, off Istria / modern-day Croatia, and were named HADR. Lastly, two large (2.75 m FL, fork length) specimens were sampled that originated from tuna traps in the Bosporus, Istanbul, Turkey in 1941, named HBOS. All specimens represented adult individuals, for an account of ages and sizes, see Riccioni et al.^[11](https://paperpile.com/c/8ySmCE/LIMRh)^.

4^th^-15^th^ century CE Yenikapi

67 vertebrae specimens were selected for analyses from a rescue excavation at a Byzantine site in the Yenikapi neighbourhood of Istanbul, Turkey. The Port of Theodosius operated at this site from 4-11^th^ century CE before being filled in at the 16^th^ century CE. The specimens used herein are conservatively dated by stratigraphic unit, archaeological context and according to the carbon dating of other specimens from the location^[43](https://paperpile.com/c/8ySmCE/P90YW)^. The specimens consist of medium to large adult individuals (~2m FL), though thorough morphological analysis is yet to be conducted. It is unknown whether the vertebrae were transported to the site from other regions, or were caught locally in the Bosporus, which supported a large Greek and Byzantine fishery. There is a potential for specimens to originate from different events by deposition in the harbour by way of the Lycus River from the city proper^[43](https://paperpile.com/c/8ySmCE/P90YW)^. See Puncher et al.^[104](https://paperpile.com/c/8ySmCE/l6gvj)^ for further details.

2^nd^ century BCE - 5^th^ century CE Baelo Claudia

55 vertebrae specimens were analysed from the Roman-era city of Baelo Claudia, Andalusia, Spain). Using the archaeological context of stratigraphic units, a total of 10 specimens were dated to the Republican Rome era (2^th^ century BCE - 1^st^ century CE) and 45 samples were dated to the Imperial Rome era (1^st^-5^th^ century CE) from various stratigraphic units and contexts within the city, predominantly associated with the fish processing facilities–called *cetariae*^[44](https://paperpile.com/c/8ySmCE/NYA3)^. Specimens represented medium sized adult individuals (~1.5m FL). The Strait of Gibraltar supported large-scale fisheries for BFT from the Phoenician era (~8^th^ century BCE) onwards, and thus specimens are believed to have been caught locally.

2^nd^ century BCE Tavira

A total of 10 vertebrae representing medium sized adults (~1.5m FL) were analysed from excavations at a Republican Rome (2^nd^ century BCE) site in Tavira, Algarve, Portugal. The archaeological context for this material is contained within an unpublished belonging to one of the co-authors, A. M-M. The vertebrae are dated according to stratigraphic units.

4^th^-2^nd^ century BCE Palacio de Justicia

Four specimens were sampled from excavations at the Cadiz site ‘Palacio de Justicia’, which was a Punic and Roman era (4^th^-2^nd^ century BCE) Palace, in Andalusia, Spain. Specimens represent medium sized adults believed to have been captured locally^[45](https://paperpile.com/c/8ySmCE/PIOwa)^.

Supplementary Figures

43

Figure S1. Scatterplot of the three outliers (black circles <-1.5 log) identified with BayeScan; SNP41, SNP43 and SNP89.


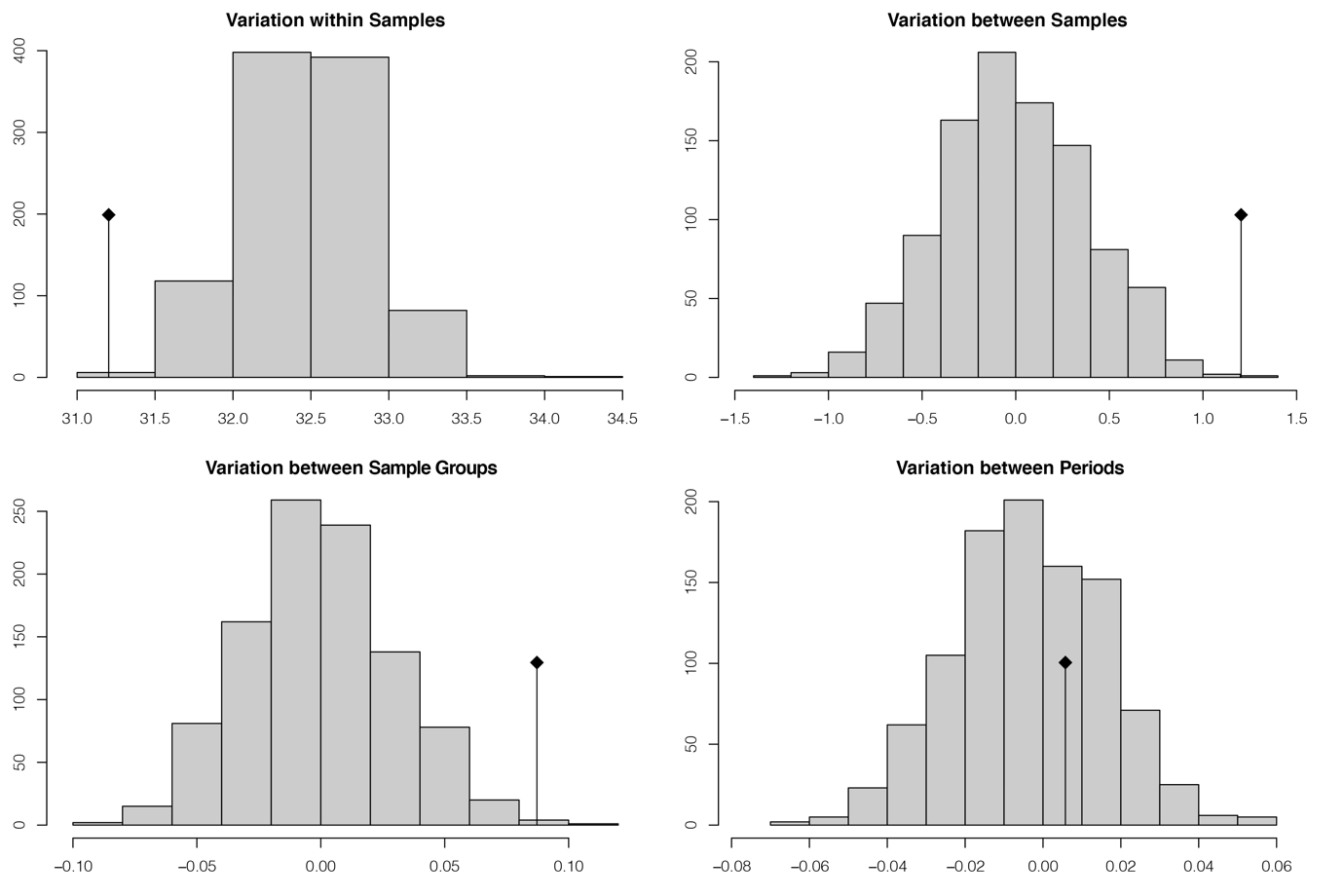


Figure S2. Barplots of variance estimated using AMOVAs using a hierarchical approach as indicated by the four levels; within samples, between samples, between sample groups and between periods (i.e. historical and contemporary). The black line indicates observed data in comparison with the expected variance from simulations (grey bars).


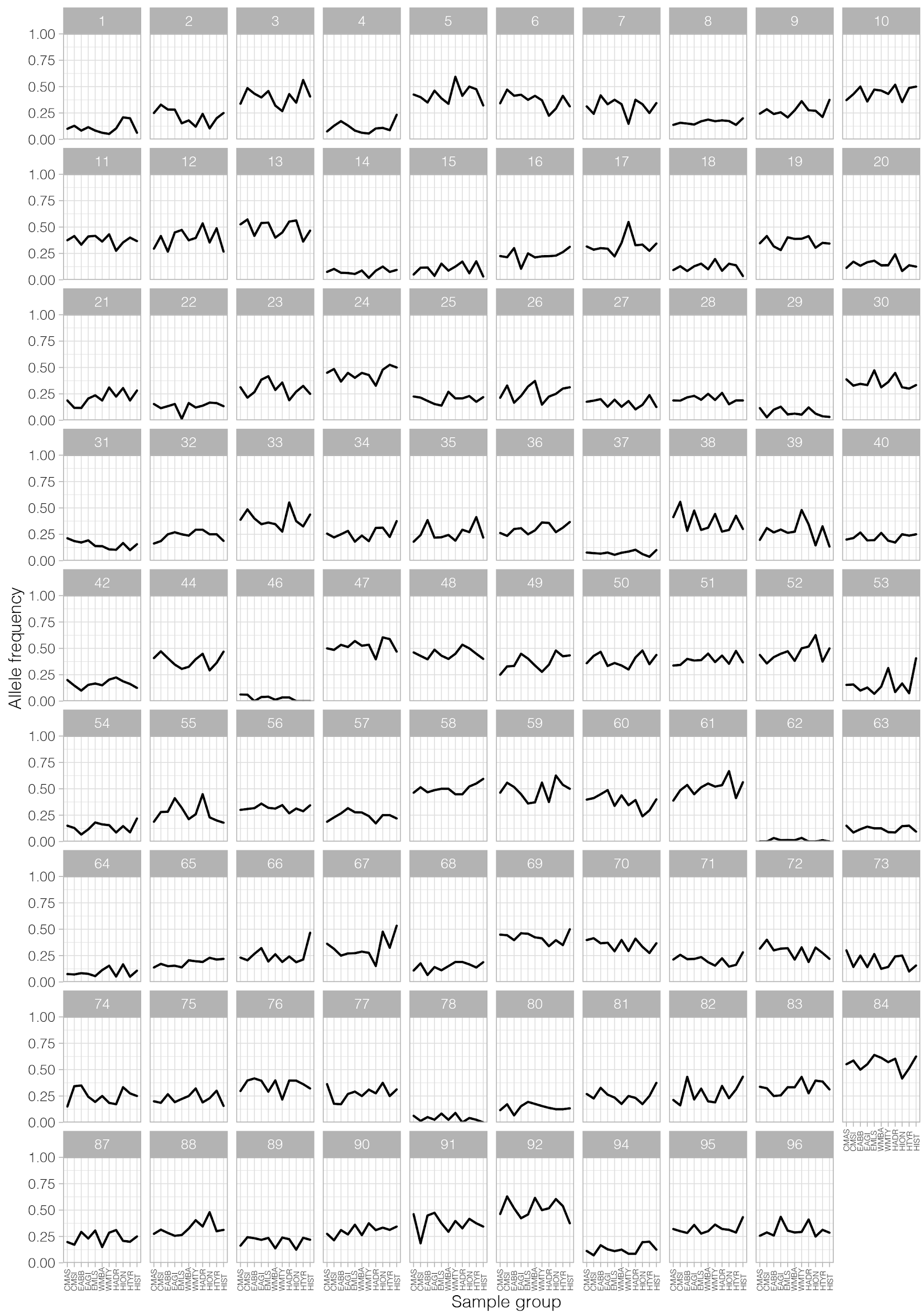


Figure S3. Line trajectory plot of minor allele frequencies per locus (excluding SNP41, SNP43 and SNP89 identified as outliers) as calculated with the round robin approach. Frequencies course across each sample group from contemporary (left sided) progressing to the earliest sample group (right sided).

Supplementary References

104. [Puncher, G. N., Onar, V., Toker, N. Y. & Tinti, F. A multitude of byzantine era bluefin tuna and swordfish bones uncovered in Istanbul, Turkey. *Collect. Vol. Sci. Pap. ICCAT/Recl. Doc. Sci. CICTA/Colecc. Doc. Cient. CICAA* **71**, 1626–1631 (2015).](http://paperpile.com/b/tVYhas/nStC)

Supplementary Tables

| Table S1. Details of contemporary, and historical (archived and archaeological) bluefin tuna (*Thunnus thynnus*) specimens collected, genotyped on the 96 loci panel, and included in the final dataset i.e. successfully genotyped. Genotype numbers of historical (archival and archaeological) samples represent those genotyped at two separate facilities to assess consistency. | | | | | | | | |
| --- | --- | --- | --- | --- | --- | --- | --- | --- |
| Sampling Location | **n sampled** | **n genotyped** | **n**  **in final dataset** | **ID** | **Age class** | **Year** | **Type** | **Reference** |
| Gulf of Mexico | 24 | 24 | 24 | GOM | YOY | 2009 | Contemporary | 14 |
| Central Mediterranean - Adriatic Sea | 40 | 40 | 40 | CMAS | Juvenile | 2011 | Contemporary | 14 |
| Central Mediterranean - Southern Sicily | 38 | 38 | 38 | CMSI | YOY | 2012 | Contemporary | 14 |
| Eastern Atlantic - Bay of Biscay | 40 | 40 | 40 | EABB | Juvenile | 2011 | Contemporary | 14 |
| Eastern Atlantic - Strait of Gibraltar | 40 | 40 | 40 | EAGI | Adult | 2011 | Contemporary | 14 |
| Eastern Mediterranean - Levantine Sea | 29 | 29 | 29 | EMLS | YOY | 2011 | Contemporary | 14 |
| Western Mediterranean - Balearic Islands | 40 | 40 | 40 | WMBA | YOY | 2011 | Contemporary | 14 |
| Western Mediterranean - Tyrrhenian Sea | 40 | 40 | 40 | WMTY | YOY | 2012 | Contemporary | 14 |
| Bosporus,  Istanbul, Turkey | 2 | 2 | 1 | HBOS | Adult | 1941 | Massimo Sella  Archive | 11 |
| Adriatic Sea,  Istria, Croatia | 49 | 49 | 21 | HADR | Adult | 1927 | Massimo Sella  Archive | 11 |
| Ionian Sea,  Zliten, Libya | 46 | 46 | 43 | HION | Adult | 1926 | Massimo Sella  Archive | 11 |
| Tyrrhenian Sea, Pizzo, Italy | 50 | 48 | 39 | HTYR | Adult | 1911 | Massimo Sella  Archive | 11 |
| Yenikapi, Istanbul, Turkey | 67 | 66 | 38 | HIST | Adult | 4^th^-15^th^ CE | Archaeological | 43 |
| Baelo Claudia, Spain | 4 | 4 | 0 | HBC | Adult | 5^th^ CE | Archaeological | 44 |
| Tavira, Portugal | 10 | 10 | 2 | HTAV | Adult | 2^nd^ BCE | Archaeological | Roselló & Morales unpublished report |
| Baelo Claudia, Spain | 45 | 45 | 0 | HBC | Adult | 2^nd^ BCE | Archaeological | 44 |
| Baelo Claudia, Spain | 6 | 6 | 0 | HBC | Adult | 2^nd^-1^st^ BCE | Archaeological | 44 |
| Palacio de Justicia, Cadiz, Spain | 4 | 4 | 0 | HPJ | Adult | 4^th^-2^nd^ BCE | Archaeological | 45 |
| n = number of individuals or archival/archaeological vertebrae  Reference indicates the primary reports detailing archival and archaeological specimens or where contemporary specimens were collected  YOY = Young-of-the-year | | | | | | | |  |

| Table S2. The 96 loci genotyped and reasons behind their selection and association as inferred from NCBI Blastn analysis | | | | | | | |
| --- | --- | --- | --- | --- | --- | --- | --- |
| SNP ID | **Reason for selection** | **Associated Protein** | **Taxa alignment** | **% identity** | **% cover** | **E-value** | **NCBI Reference code** |
| 1 | *Polymorphic between contemporary populations in Puncher et al.^14^* |  |  |  |  |  |  |
| 2 | *Gene association* | Phosphoglucomutase 1 | *Dicentrarchus labrax* | 95.92 | 80.99 | 6.00E-39 | LG4:6467652-6467749 |
| 3 | *Gene association* | Myoglobin | *teleosts* | 100 | 81.82 | 3.00E-11 | E1CPX3_THUMA |
| 4 | *Gene association* |  |  |  |  |  |  |
| 5 | *Gene association* | Vacuolar protein sorting-associated protein 28 homolog | *Dicentrarchus labrax* | 91.74 | 100 | 1.00E-39 | LG9:13694258-13694378 |
| 5 | *Gene association* | Vacuolar protein sorting-associated protein 28 homolog | *teleosts* | 94.29 | 86.78 | 3.00E-11 | C1BLI6_OSMMO |
| 6 | *Gene association* | Betaine homocysteine methyltransferase isoform 3 OS | *teleosts* | 97.14 | 86.78 | 3.00E-12 | V9HXV7_SPAAU |
| 7 | *Gene association* | Amylo-6-4-alpha-glucanotransferase isoform 1 | *Dicentrarchus labrax* | 84.03 | 98.35 | 3.00E-16 | LG10:1491505-1491623 |
| 8 | *Polymorphic between contemporary populations in Puncher et al.^14^* |  |  |  |  |  |  |
| 9 | *Polymorphic between contemporary populations in Puncher et al.^14^* |  |  |  |  |  |  |
| 10 | *Polymorphic between contemporary populations in Puncher et al.^14^* |  |  |  |  |  |  |
| 11 | *Polymorphic between contemporary populations in Puncher et al.^14^* |  |  |  |  |  |  |
| 12 | *Polymorphic between contemporary populations in Puncher et al.^14^* |  |  |  |  |  |  |
| 13 | *Polymorphic between contemporary populations in Puncher et al.^14^* |  |  |  |  |  |  |
| 14 | *Gene association* | Phosphoglucomutase 1 | *Dicentrarchus labrax* | 90.6 | 96.69 | 1.00E-33 | LG4:6464452-6464568 |
| 15 | *Gene association* | RUVb-like 1-like | *Dicentrarchus labrax* | 85.58 | 85.95 | 3.00E-16 | LG1A:15592644-15592747 |
| 16 | *Gene association* | Betaine-homocysteine s-methyltransferase 1-like | *Dicentrarchus labrax* | 89.38 | 93.39 | 8.00E-32 | LG20:4801689-4801801 |
| 16 | *Gene association* | Betaine homocysteine methyltransferase isoform 4 | *teleosts* | 92.31 | 96.69 | 5.00E-16 | V9HZ12_SPAAU |
| 17 | *Gene association* | T-complex protein 1 subunit epsilon | *Dicentrarchus labrax* | 91.8 | 100 | 4.00E-37 | LG18-21:6432507-6432628 |
| 18 | *Gene association* | Trifunctional enzyme subunit mitochondrial | *Dicentrarchus labrax* | 92.31 | 96.69 | 6.00E-39 | LG17:12286073-12286189 |
| 18 | *Gene association* | Hydroxyacyl-CoA dehydrogenase/3-ketoacyl-CoA thiolase/enoyl-CoA hydratase (trifunctional protein), beta subunit | *Gadus morhua* | 95 | 99.17 | 2.00E-18 | ENSGMOP00000019175 |
| 18 | *Gene association* | Hydroxyacyl-Coenzyme A dehydrogenase | *teleosts* | 97.5 | 99.17 | 2.00E-17 | G1FKK0_EPIBR |
| 19 | *Gene association* | Calumenin isoform x2 | *Dicentrarchus labrax* | 85.09 | 94.21 | 2.00E-17 | UN:33920969-33921082 |
| 20 | *Polymorphic between contemporary populations in Puncher et al.^14^* |  |  |  |  |  |  |
| 21 | *Polymorphic between contemporary populations in Puncher et al.^14^* |  |  |  |  |  |  |
| 22 | *Polymorphic between contemporary populations in Puncher et al.^14^* |  |  |  |  |  |  |
| 23 | *Polymorphic between contemporary populations in Puncher et al.^14^* |  |  |  |  |  |  |
| 24 | *Polymorphic between contemporary populations in Puncher et al.^14^* |  |  |  |  |  |  |
| 25 | *Polymorphic between contemporary populations in Puncher et al.^14^* |  |  |  |  |  |  |
| 26 | *Gene association* | Xin actin-binding repeat-containing protein 2-like | *Dicentrarchus labrax* | 88.03 | 96.69 | 2.00E-26 | LG15:12489701-12489817 |
| 27 | *Gene association* | M-protein, striated muscle | *teleosts* | 82.05 | 96.69 | 2.00E-11 | W5UM88_ICTPU |
| 28 | *Gene association* | Reticulon-4-like isoform 1 | *Dicentrarchus labrax* | 94.12 | 98.35 | 6.00E-42 | LG11:22112661-22112778 |
| 29 | *Gene association* | SET and MYND domain containing protein 1a | *teleosts* | 85 | 99.17 | 4.00E-15 | W0M2F7_SINCH |
| 30 | *Gene association* | Myozenin 1 | *Dicentrarchus labrax* | 91.92 | 81.82 | 5.00E-30 | LG1B:12030692-12030790 |
| 31 | *Gene association* | COP9 constitutive photomorphogenic homolog subunit 5 | *Gadus morhua* | 100 | 84.3 | 2.00E-13 | ENSGMOP00000018264 |
| 31 | *Gene association* | COP9 signalosome complex subunit 5 | *teleosts* | 100 | 84.3 | 5.00E-12 | C1BF76_ONCMY |
| 32 | *Polymorphic between contemporary populations in Puncher et al.^14^* |  |  |  |  |  |  |
| 33 | *Polymorphic between contemporary populations in Puncher et al.^14^* |  |  |  |  |  |  |
| 34 | *Polymorphic between contemporary populations in Puncher et al.^14^* |  |  |  |  |  |  |
| 35 | *Polymorphic between contemporary populations in Puncher et al.^14^* |  |  |  |  |  |  |
| 36 | *Polymorphic between contemporary populations in Puncher et al.^14^* |  |  |  |  |  |  |
| 37 | *Polymorphic between contemporary populations in Puncher et al.^14^* |  |  |  |  |  |  |
| 38 | *Gene association* | Eukaryotic translation initiation factor 3 subunit g | *Dicentrarchus labrax* | 93.39 | 100 | 2.00E-44 | LG8:11023666-11023786 |
| 39 | *Gene association* | Proline-rich nuclear receptor coactivator 2 | *Dicentrarchus labrax* | 88.24 | 98.35 | 8.00E-26 | LG16:4979463-4979579 |
| 40 | *Gene association* | Thioredoxin interacting protein | Thunnus thynnus | 100 | 100 | 3.00E-64 | EC918814 |
| 40 | *Gene association* | Thioredoxin-interacting protein | *Dicentrarchus labrax* | 89.66 | 95.87 | 1.00E-30 | LG9:3159022-3159137 |
| 40 | *Gene association* | Thioredoxin-interacting protein | *teleosts* | 97.5 | 99.17 | 5.00E-16 | I7HH00_OPLFA |
| 41 | *Gene association* | Synemin isoform x1 | *Dicentrarchus labrax* | 85.95 | 100 | 1.00E-21 | LG6:9865641-9865761 |
| 42 | *Gene association* | Complement factor d-like | *Dicentrarchus labrax* | 89.52 | 100 | 8.00E-32 | LG12:21422927-21423050 |
| 42 | *Gene association* | Complement factor D (adipsin) | *Gadus morhua* | 82.5 | 96.69 | 3.00E-15 | ENSGMOP00000005890 |
| 42 | *Gene association* | Kallikrein like protein | *teleosts* | 85 | 96.69 | 4.00E-15 | Q2Z1R4_ORYLA |
| 43 | *Gene association* | Ryanodine receptor 3-like | *Dicentrarchus labrax* | 86.73 | 93.39 | 2.00E-20 | LG17:12874139-12874248 |
| 44 | *Polymorphic between contemporary populations in Puncher et al.^14^* |  |  |  |  |  |  |
| 45 | *Polymorphic between contemporary populations in Puncher et al.^14^* |  |  |  |  |  |  |
| 46 | *Polymorphic between contemporary populations in Puncher et al.^14^* |  |  |  |  |  |  |
| 47 | *Polymorphic between contemporary populations in Puncher et al.^14^* |  |  |  |  |  |  |
| 48 | *Polymorphic between contemporary populations in Puncher et al.^14^* |  |  |  |  |  |  |
| 49 | *Gene association* | Myosin heavy chain | *teleosts* | 87.18 | 96.69 | 6.00E-13 | Q98TQ4_9TELE |
| 50 | *Gene association* | Muscle-specific beta 1 integrin binding protein 2 | *teleosts* | 85.71 | 86.78 | 2.00E-11 | B9V3X3_EPICO |
| 51 | *Gene association* | Platelet-derived growth factor receptor-like | Thunnus thynnus | 93.97 | 94.21 | 1.00E-38 | EH000371 |
| 52 | *Gene association* | Heat shock protein 25 variant 1 | *Dicentrarchus labrax* | 90.74 | 89.26 | 1.00E-30 | LG8:21804879-21804986 |
| 53 | *Gene association* | Homeobox protein tgif2 | *Dicentrarchus labrax* | 83.33 | 99.17 | 1.00E-12 | LG1A:12106316-12106433 |
| 54 | *Gene association* | High-mobility group box 1 | Thunnus thynnus | 100 | 93.39 | 2.00E-59 | EC919262 |
| 54 | *Gene association* | High mobility group-t | *Dicentrarchus labrax* | 92.37 | 97.52 | 6.00E-39 | LG14:17296613-17296730 |
| 55 | *Polymorphic between contemporary populations in Puncher et al.^14^* |  |  |  |  |  |  |
| 56 | *Polymorphic between contemporary populations in Puncher et al.^14^* |  |  |  |  |  |  |
| 57 | *Polymorphic between contemporary populations in Puncher et al.^14^* |  |  |  |  |  |  |
| 58 | *Polymorphic between contemporary populations in Puncher et al.^14^* |  |  |  |  |  |  |
| 59 | *Polymorphic between contemporary populations in Puncher et al.^14^* |  |  |  |  |  |  |
| 60 | *Polymorphic between contemporary populations in Puncher et al.^14^* |  |  |  |  |  |  |
| 61 | *Gene association* | Sarcoglycan delta | *Dicentrarchus labrax* | 90.83 | 97.52 | 8.00E-29 | LG14:23358400-23358517 |
| 62 | *Gene association* | Cyclic amp-dependent transcription factor atf-4-like | *Dicentrarchus labrax* | 93.33 | 86.78 | 1.00E-33 | LG7:27011773-27011876 |
| 63 | *Gene association* | Platelet-derived growth factor receptor-like | *Dicentrarchus labrax* | 84.35 | 95.04 | 1.00E-15 | LG2:14696671-14696785 |
| 64 | *Gene association* | Ttnl protein | *teleosts* | 83.33 | 89.26 | 4.00E-09 | A0JMJ0_DANRE |
| 65 | *Gene association* | Cofilin-2 | *Dicentrarchus labrax* | 95.76 | 97.52 | 2.00E-48 | LG2:5701470-5701587 |
| 66 | *Gene association* | Myeloid leukemia factor 1-like | *Dicentrarchus labrax* | 84.3 | 100 | 7.00E-17 | LG13:16731815-16731935 |
| 67 | *Polymorphic between contemporary populations in Puncher et al.^14^* |  |  |  |  |  |  |
| 68 | *Polymorphic between contemporary populations in Puncher et al.^14^* |  |  |  |  |  |  |
| 69 | *Polymorphic between contemporary populations in Puncher et al.^14^* |  |  |  |  |  |  |
| 70 | *Polymorphic between contemporary populations in Puncher et al.^14^* |  |  |  |  |  |  |
| 71 | *Polymorphic between contemporary populations in Puncher et al.^14^* |  |  |  |  |  |  |
| 72 | *Polymorphic between contemporary populations in Puncher et al.^14^* |  |  |  |  |  |  |
| 73 | *Gene association* | Myomesin-1 | *teleosts* | 82.5 | 99.17 | 1.00E-11 | E6ZJ46_DICLA |
| 74 | *Gene association* | Phosphorylase kinase gamma subunit 1 | *Dicentrarchus labrax* | 87.63 | 80.17 | 3.00E-19 | LG13:15806738-15806834 |
| 75 | *Gene association* |  |  |  |  |  |  |
| 76 | *Gene association* | Smoothelin-like protein 2-like | *Dicentrarchus labrax* | 89.74 | 96.69 | 3.00E-31 | LG14:26383715-26383831 |
| 77 | *Gene association* | Elongation factor 2 | *Dicentrarchus labrax* | 93.52 | 89.26 | 9.00E-38 | LG10:10016955-10017062 |
| 77 | *Gene association* | Eukaryotic translation elongation factor2 | *Gadus morhua* | 82.5 | 99.17 | 5.00E-14 | ENSGMOP00000012542 |
| 77 | *Gene association* | Eukaryotic translation elongation factor 2, like | *teleosts* | 95 | 99.17 | 1.00E-15 | Q7ZVM3_DANRE |
| 78 | *Gene association* | Elongation factor 1 alpha | Thunnus thynnus | 95 | 99.17 | 6.00E-47 | EL610929 |
| 78 | *Gene association* | Elongation factor 1-alpha | *Dicentrarchus labrax* | 97.5 | 99.17 | 2.00E-54 | LG9:21157592-21157711 |
| 78 | *Gene association* | Elongation factor 1 alpha | *Gadus morhua* | 90 | 99.17 | 7.00E-16 | ENSGMOP00000012846 |
| 78 | *Gene association* | Elongation factor 1-alpha | *teleosts* | 100 | 99.17 | 1.00E-16 | A0A087XDA8_POEFO |
| 79 | *Polymorphic between contemporary populations in Puncher et al.^14^* |  |  |  |  |  |  |
| 80 | *Polymorphic between contemporary populations in Puncher et al.^14^* |  |  |  |  |  |  |
| 81 | *Polymorphic between contemporary populations in Puncher et al.^14^* |  |  |  |  |  |  |
| 82 | *Polymorphic between contemporary populations in Puncher et al.^14^* |  |  |  |  |  |  |
| 83 | *Polymorphic between contemporary populations in Puncher et al.^14^* |  |  |  |  |  |  |
| 84 | *Polymorphic between contemporary populations in Puncher et al.^14^* |  |  |  |  |  |  |
| 85 | *Gene association* | Phosphoglucomutase 1 | *Dicentrarchus labrax* | 86.09 | 94.21 | 4.00E-18 | LG4:6461135-6461249 |
| 86 | *Gene association* | Keratin 23 (histone deacetylase inducible | *Gadus morhua* | 94.59 | 91.74 | 1.00E-13 | ENSGMOP00000015062 |
| 87 | *Gene association* | Protein kinase c inhibitor aswz variant 5 | *Dicentrarchus labrax* | 88.46 | 85.95 | 5.00E-24 | LG11:15886279-15886382 |
| 87 | *Gene association* | Histidine triad nucleotide-binding protein | *teleosts* | 82.86 | 86.78 | 2.00E-09 | E3TGF9_ICTPU |
| 87 | *Gene association* | Protein FAM171b-like | *Dicentrarchus labrax* | 90 | 100 | 1.00E-132 | LG24:2370971-2371370 |
| 88 | *Gene association* | Myozenin 1 | *Dicentrarchus labrax* | 96.69 | 100 | 1.00E-52 | LG1B:12032167-12032287 |
| 88 | *Gene association* | Myozenin-2 | *teleosts* | 82.5 | 99.17 | 5.00E-12 | E3TCH3_ICTFU |
| 89 | *Gene association* | Calponin 1, basic, smooth muscle | *Gadus morhua* | 91.67 | 89.26 | 3.00E-13 | ENSGMOP00000016108 |
| 89 | *Gene association* | Calponin | *teleosts* | 88.89 | 89.26 | 4.00E-12 | M4A2Z2_XIPMA |
| 90 | *Gene association* | Alpha cardiac-like isoform 1 | *Dicentrarchus labrax* | 93.44 | 100 | 2.00E-41 | LG12:9448943-9449064 |
| 91 | *Polymorphic between contemporary populations in Puncher et al.^14^* |  |  |  |  |  |  |
| 92 | *Polymorphic between contemporary populations in Puncher et al.^14^* |  |  |  |  |  |  |
| 93 | *Polymorphic between contemporary populations in Puncher et al.^14^* |  |  |  |  |  |  |
| 94 | *Polymorphic between contemporary populations in Puncher et al.^14^* |  |  |  |  |  |  |
| 95 | *Polymorphic between contemporary populations in Puncher et al.^14^* |  |  |  |  |  |  |
| 96 | *Polymorphic between contemporary populations in Puncher et al.^14^* |  |  |  |  |  |  |

| Table S3. Effective population size (N_e_) and 95% Confidence Intervals of contemporary and historical sample groups for samples (n) consistently scored across all 89 neutral loci analysed herein, under two approaches, where separate estimates were made for each sample group and for contemporary and historical pools. The number of samples analysed (n) per sample group were equalised by random down-sampling. | | | | | | | | | | | | | |
| --- | --- | --- | --- | --- | --- | --- | --- | --- | --- | --- | --- | --- | --- |
|  |  | **Contemporary** | | | | | | | | **Historical** | | | |
|  |  | **GOM** | **CMAS** | **CMSI** | **EABB** | **EAGI** | **EMLS** | **WMBA** | **WMTY** | **HADR** | **HION** | **HTYR** | **HIST** |
| **Separate** | **n** | 14 | 14 | 14 | 14 | - | 14 | 14 | 14 | - | 14 | 14 | 14 |
|  | **N_e_** | 154 | 93 | 175 | ∞ | - | 806 | 140 | ∞ | - | ∞ | 26 | 19 |
|  | **CI** | 47 - ∞ | 38 - ∞ | 48 - ∞ | 76.7 - ∞ | - | 64 - ∞ | 46 - ∞ | 98 - ∞ | - | 244 - ∞ | 18 - 45 | 14 - 29 |
| **Pooled** | **n** | 0 | 14 | 15 | 17 | 0 | 2 | 7 | 15 | 4 | 24 | 24 | 18 |
|  | **N_e_** |  | 1049 | | | | | | |  | 285 | | |
|  | **CI** |  | 321 - ∞ | | | | | | |  | 173 - 716 | | |
